# Postpartum family planning utilization in Burundi and Rwanda: a comparative analysis of population based cross-sectional data

**DOI:** 10.11604/pamj.2018.30.303.15105

**Published:** 2018-08-31

**Authors:** Gideon Rutaremwa, Allen Kabagenyi

**Affiliations:** 1 United Nations Economic Commission for Africa (UNECA) Addis Ababa, Ethiopia; 2 Department of Population Studies, School of Statistics and Planning, College of Business and Management Sciences, Makerere University, Kampala, Uganda

**Keywords:** Postpartum, family planning, contraception, utilization, Burundi, Rwanda, women

## Abstract

**Introduction:**

Promotion of modern family planning is a major policy action for Africa to harness the demographic dividend. Family planning is an important public health intervention for maternal and child health.

**Methods:**

Analysis was based on data from Demographic and Health Surveys conducted in 2010 on samples of women from Burundi (3396) and Rwanda (4670). Descriptive and multivariate logistic regression analyses to examine the contribution and comparison of the various predictors of uptake of modern contraceptives during the postpartum period (PPFP) in the two countries were carried out using STATA statistical software.

**Results:**

Descriptive findings show only 20% of the samples of women in Burundi used while more than half of the women (51%) were using PPFP. Utilization of PPFP was significantly associated with primary (OR = 1.3, 95% CI = 1.1-1.6) and higher education (OR = 2.2, 95% CI = 1.6-3.1) in Burundi. Similarly in Rwanda increased use of PPFP in primary was (OR = 1.4, 95% CI = 1.2-1.6) while secondary education (OR = 1.6, 95% CI = 1.2-2.1). Protestant women were less likely to use PPFP in both Burundi (OR = 0.75, 95% CI = 0.6-0.9) and Rwanda (OR = 0.69, 95% CI = 0.6-0.8). Other significant variables in the regression models of both countries included wealth status, age of woman, number of living children and exposure to media. Professional birth delivery assistance was significant only in Rwanda.

**Conclusion:**

Enhancing postpartum contraceptive use should target women with low education, low wealth status, and that the media has an important role to play in this transformation. Policies and programs must be put in place to ensure that the rural urban differences are eradicated.

## Introduction

Expanding access to family planning is an effective strategy for saving women's and children's lives and improving their health [1]. Family planning empowers women and households to make decisions about whether and when to have children as well as the desired family size. For Africa to harness the demographic dividend, one of the key initiatives that have been identified as a major policy action is the promotion of modern family planning [2]. The 1994 International Conference on Population and Development held in Cairo called for a radical change in programs away from demographic aims and toward reproductive health and equally called for improvement of the situation of women and noted that the future family planning frontier will be sub-Saharan Africa, for which radically new types of programs may have to be developed [3]. The postpartum period is a time when many routine interventions are provided to mothers, besides during the postpartum period, most mothers want to delay or stop the next pregnancy [4]. Uptake of postpartum family planning (PPFP) remains low in Eastern Africa and very little is known about how pregnant women arrive at their decisions to adopt PPFP. Yet, the benefit of early adoption or continuation of family planning are well known and are positive [5]. Providing effective, high quality antenatal and post-partum contraceptive counseling can reduce unintended pregnancies, decrease maternal and fetal morbidity and mortality and prevent unsafe abortions. In order to improve the situation, health authorities should be encouraged to provide counseling on postpartum contraceptive methods during antenatal care (ANC) and immediate postpartum period [6]. The postpartum period is a critical time to address high unmet family planning need and to reduce the risks of closely spaced pregnancies [7-9].

Postpartum family planning is defined as the prevention of unintended pregnancy and closely spaced pregnancies through the first 12 months following childbirth [8]. A study in Nepal revealed that more than one-quarter of women who gave birth in the last five years became pregnant within 24 months of giving birth and 52% had an unmet need for family planning within 24 months postpartum [10]. The rationale for promotion of family planning to delay conception after a recent birth is a best practice that can lead to optimal maternal and child health outcomes [5, 8, 11, 12]. On the contrary, short inter-pregnancy intervals can result in negative health outcomes such as maternal anaemia, low birth weight and neonatal and infant mortality [8, 13, 14]. Evidence suggests that some women have become pregnant early in the postpartum period [4, 15], given that there is a resumption of sexual activity which exposes women to conception [7]. A study of postpartum ovulation and menstruation found that ovulation occurred prior to menstruation in 20 percent of the women who bottle-fed their babies and in 45 percent of breast feeders [16]. Such evidence have led many to argue that it is better to initiate contraception immediately after childbirth than to incur the risk of unprotected intercourse [17]. The latter has made to recommendation that practical tools be included in the resource for integrating postpartum family planning at points when women have frequent health system contact, including antenatal care, labor and delivery, postnatal care, immunization, and child health care [9].

Recent demographic and health survey data show that total fertility rates in Eastern African Sub-Region were highest in Burundi at 6.4 [18] and were lowest in Rwanda at 4.6 [19, 20] children, respectively. The low fertility rates in Rwanda have been explained to be a result of improvements to Rwanda´s health system, infrastructure, and social programs over the last decade which has led to a rapid fertility transition unique from other African countries. The total fertility rate fell from 6.1 in 2005 to 4.6 in 2010, with a 3-fold increase in contraceptive prevalence rates [20]. In Burundi on the other hand fertility rates have remained very high, a situation that could be attributed to the strong cultural and societal pressures on women to bear more children [21]. One of the intriguing issues in this study was how two communities with seemingly comparable historical, socioeconomic and geo-political setting could be at much varying pace of demographic transition. Obviously, one of the likely explanations that was explored in this study is utilization of family planning during the postpartum period. The key question addressed by this study is therefore: what are the predictors of modern contraceptive use during the post-partum period in Burundi and Rwanda? The current study therefore addresses the following two specific objectives: first, it sought to establish the level of contraceptive use during the postpartum period in Burundi and Rwanda; and second, analyses the predictors of PPFP in in the two study populations. Such information would be necessary to establish postpartum family planning services where they are truly responsive to the desires of potential clients [15].

## Methods

**Data source:** We used secondary population-based cross-sectional survey data-the Demographic and Health Surveys (DHS) conducted in 2010 in both Burundi [18] and Rwanda [19]. The DHS employed nationally representative samples, which were based on a two-stage stratified sample of households. These types of surveys are carried out in order to provide information on national demographic, socio-economic, health indicators including contraceptive use among women. These data can easily accessed on approval from the measuredhs website. The DHS employed nationally representative samples, which were based on a two-stage stratified sample of households. These types of surveys are carried out every after five years to provide information on national demographic, socio-economic, health indicators including contraceptive use among women. A total of 3396 and 4670 women from Burundi and Rwanda respectively were selected for the current study. The samples comprised of women who had completed the full 12 months of the postpartum period. Only the most recent birth was considered, even if a woman delivered more than once during the reference period. We excluded women who did not have a live birth during the five years before the survey.

**Variables:** We defined postpartum contraceptive or post-partum family planning (PPFP) as a woman's use of any modern method of contraception during the 12 months following her most recent childbirth. The modern methods included pill, IUD, injection, diaphragm, condom (male or female), sterilization (male or female), implant, or foam/jelly). Therefore, the outcome variable in this study-PPFP, was expressed as the binary outcome, whether a woman utilized modern postpartum family planning or otherwise. PPFP was coded 1, if a woman used any modern method of contraception and coded 0 otherwise. Predictor variables considered (Table 1) included those commonly reported in the contraceptive use studies. They included (categories are given in parentheses): place of residence (urban, rural), education level attainment (no education, primary, secondary and higher), religion (Catholic, Protestant, Muslims, others; where other religions include Seventh Day Adventists (SDA) and unknown religions), wealth index (poorest, poor middle, rich and richest), marital status (never married, currently married, previously married). Additional variables included the following: age of the woman (continuous), number of surviving children, exposure to media (whether a woman had ever accessed family planning information through print, radio and television channels), skilled birth attendance and timing of post-delivery check-up.

**Table 1:** Percentage distribution of the sample population by selected backgroundpredictor variables

Variable/Category	Burundi		Rwanda	
	Number	Percent	Number	Percent
**Educational Level attainment**				
None	1,831	53.9	908	19.4
Primary	1,347	39.7	3320	71.1
Secondary	219	6.4	442	9.5
**Religion**				
Catholic	2,090	61.5	1952	41.8
Protestant	1,118	32.9	1974	42.3
Muslim	91	2.7	653	14.0
All other	98	2.9	91	2.0
**Age group**				
15-19	56	1.7	52	1.1
20-24	636	18.7	691	14.8
25-29	879	25.9	1334	28.6
30-34	629	18.5	1053	22.6
35-39	651	19.2	777	16.6
40-44	361	10.6	496	10.6
45-49	184	5.4	268	5.7
**Residence**				
urban	303	8.9	608	13.0
rural	3,093	91.1	4062	87.0
**Wealth Quintile**				
Poorest	746	22.0	1072	23.0
Poorer	751	22.1	965	20.7
Middle	681	20.1	927	19.9
Richer	634	18.7	886	19.0
Richest	584	17.2	820	17.6
**Exposure to media**				
No	1,905	56.1	1456	31.2
Yes	1,491	43.9	3214	68.8
**Delivery Assistance**				
None Professional	1,217	35.8	1411	30.2
Professional	2,179	64.2	3259	69.8
**Timing of postnatal care**				
<1 day	190	5.6	162	3.5
1-2 days	83	2.4	71	1.5
3+ days	131	3.9	97	2.1
Other	2,992	88.1	4340	92.9
**Postpartum Family Planning use**				
No	2,735	80.6	2286	49.0
Yes	661	19.5	2384	51.1
**Total (N)**	**3396**		**4670**	

Note: some of the totals for the respective variables do not sum up to the overall total because of the weights applied to the data

**The study sample:** A total of 3396 women from Burundi and 4670 from Rwanda were selected for this study. All women selected for this study were postpartum and were aged 15-49 years were extracted from the 2010 DHS datasets of the two countries for further analyses. Women included in the analysis, had completed the full 12 months of the postpartum period. Only the most recent birth was considered, even if a woman delivered more than once during the reference period. We excluded women who did not have a live birth in the five years preceding the survey date. In the main analysis, we applied both descriptive analyses (Table 1) and later multivariate analysis using a binary logistic regression model (Table 2) to analyze the relative contribution of the various predictors of uptake of modern contraceptives during the postpartum period.

**Table 2:** Logistic regression model predicting the odds of utilizing postpartum family planning in Burundi (Model 1) and Rwanda (Model 2)

		Model I (Burundi)			Model II (Rwanda)	
Variables/category	Odds Ratio	p-value	Confidence Interval	Odds Ratio	p-value	Confidence Interval
Age of woman^§^	0.94	0.00	[0.92-0.96]	0.94	0.00	[0.92-0.95]
**Education Level**						
None^®^	1. 00	.	.	1.00	.	.
Primary	1.30	0.01	[1.06-1.59]	1.40	0.00	[1.19-1.64]
Secondary	2.21	0.00	[1.59-3.07]	1.59	0.00	[1.22-2.06]
**Wealth Quintile**						
Poorest^®^	1. 00	.	.	1.00	.	.
Poorer	0.89	0.44	[0.65-1.20]	1.12	0.23	[0.93-1.34]
Middle	0.99	0.97	[0.73-1.36]	1.35	0.00	[1.12-1.62]
Richer	1.06	0.72	[0.77-1.46]	1.37	0.00	[1.13-1.65]
Richest	1.35	0.08	[0.97-1.88]	1.36	0.01	[1.09-1.70]
**Religion**						
Catholic^®^	1. 00	.	.	1.00	.	.
Protestant	0.75	0.01	[0.62-0.92]	0.69	0.00	[0.61-0.79]
Moslem	1.51	0.05	[1.00-2.27]	0.94	0.48	[0.78-1.12]
All Other	2.03	0.00	[1.27-3.23]	0.76	0.23	[0.49-1.19]
**Delivery Assistance**						
Other^®^	1.00	.	.	1.00	.	.
Health professional	1.15	0.21	[0.93-1.42]	1.18	0.02	[1.03-1.36]
**Timing of postnatal Care**						
<1 day^®^	1.00	.	.	1.00	.	.
1-2 days	0.80	0.48	[0.43-1.48]	1.37	0.27	[0.78-2.41]
3+ days	0.63	0.11	[0.35-1.11]	1.39	0.22	[0.82-2.35]
Other	0.78	0.14	[0.57-1.08]	0.96	0.80	[0.70-1.32]
**Residence**						
Urban^®^	1.00	.	.	1.00	.	.
Rural	0.76	0.04	[0.58-0.99]	1.15	0.17	[0.94-1.41]
**Exposure to media**						
None^®^	1.00	.	.	1.00	.	.
Yes	1.58	0.00	[1.31-1.89]	1.33	0.00	[1.17-1.52]
Number of living children^§^	1.28	0.00	[1.20-1.37]	1.28	0.00	[1.22-1.34]
Model Constant	0.66	0.25	[0.32-1.33]	1.90	0.02	[1.10-3.30]
		*N=3305; Pseudo R^2^=7%*			*N=4624; Pseudo R^2^=4%*	

Note: **^®^** = reference category; ^§^Age of woman and Number of living children were put in the model as continuous variables

**Statistical analysis:** Descriptive statistics (either as percentages for the categorical variables or mean for the continuous variables) are shown for selected predictors (Table 1). In the main analysis, a logistic regression model was fitted to the data for the two countries and the odd ratios (ORs) based on the 95% confidence intervals were calculated for each group of the categorical predictors (Table 2). The logistic regression models were used to predict the log-odds of using modern contraceptives during the postpartum period. Formally, the estimated equation may be expressed as follows: (1) Where; logit [P (Y = 1)] refers to the natural log odds that a respondent will: use postpartum contraception, β_0_ refers to the intercept of the regression model; and β_i_X_j_refer to regression estimates for the set of explanatory variables (numbered 1 through k) included in the models. For purposes of accounting for the complex sample design, used in DHS data collection, we weighted the data during the analysis. This procedure was intended to iron out the effects of clustering and stratification as well as the effect of sampling weights when computing the variance, standard error and confidence intervals (the *"vce-robust"* option was used). The variables were also checked for multi-collinearity, and those variables that passed the test were included in the final model. We analyzed the data using STATA software version 13.

**Ethical approval and consent to participate:** Approval for DHS data utilization for this study was obtained from the data originator, ICF Macro International U.S.A before the data was extracted from their web platform. At the point of data collection by the data originators, an informed consent was sought from all the study participants after detailed description of all the issues related to the study were passed across to the respondents. Eligible respondents who were unwilling to participate in the study were excluded from the survey. All participant information that was used in the analysis was purely anonymous based on the principle of confidentiality of participants. Detailed information about data acquisition ethical approvals can be got from www.measuredhs.com.

## Results

**Descriptive results:** The findings presented in Table 1 show that there were marked differences and similarities in the distribution of the study populations in Burundi and Rwanda, respectively. Whereas 53.9% of the study population from Burundi had no education, only 19.4% of their counterparts from Rwanda had no education. Religious distributions in the two country study samples were also fairly different. Catholics comprised 61.5% of the sample in Burundi and only 41.8% of the Rwandan sample, while Protestants comprised 32.9% and 42.3% in Burundi and Rwanda respectively. The age distributions of the two study populations were largely similar in pattern. In terms of rural urban residence characteristics, the urban proportion in Burundi sample comprised about 9% while in Rwanda it was 13%. The wealth status distribution of the two samples was nearly similar with the bottom two quintiles in both countries comprising about 44%, while the top two wealth quintiles comprised about 36% of the sample in both study populations. There were differences in the two samples with regard to exposure to media, with 56% of the sample in Burundi indicating that they had no media exposure, compared to only 31% of the sample from Rwanda. Other differences in the distribution of the characteristics of the sample were observed for the variable "assistance during delivery". Whereas only 64.2% of the Burundi sample had professional supervised delivery, in Rwanda nearly 70% had supervised delivery. Finally, the findings in Table 1 show that whereas only about20% of the sampled women in Burundi used PPFP, in Rwanda more than half of the women (51.1%) were using PPFP.

**Multivariate regression model results:** Table 2 presents results from the multivariate regression model depicting the relative odds of utilizing PPFP for both Burundi (Model 1) and Rwanda (Model 2), respectively. The findings in Table 2 show that each additional year increase in the age of woman was significantly associated with reduced odds of utilization of PPFP for both Burundi (OR = 0.94, 95% CI = 0.92-0.96) and Rwanda (OR = 0.94, 95% CI = 0.92-0.95). However, education level attainment of the woman was directly related to their utilization of PPFP services. In Burundi women with a primary level education had 1.3 times the odds of utilizing PPFP (95% CI = 1.1-1.6) compared to those who had no education, which those with a secondary and higher level had 1.6 times the odds of PPFP (95% CI = 1.6-3.1) compared to the same reference category. Similar pattern of education level attainment findings was observed for Rwanda as in Burundi. Women with a primary level education experience 1.4 times the odds of PPFP utilization (95% CI = 1.2-1.6) compared to those with no education. Women who had attained at least a secondary level education had 1.6 the odds of PPFP utilization (95% CI = 1.2-2.1) compared to those with no education. While wealth status indicator depicted significant results for Rwanda, in Burundi the results for this variable were not significant in the regression model. In Rwanda, the likelihood of utilizing PPFP was higher among women in higher wealth quintiles compared to those in the poorest category. Notably, women in the middle wealth quintile and higher experience nearly 1.4 times the odds of PPFP (middle category - 95% CI = 1.1-1.6; richer and richest categories - 95% CI = 1.1-1.7) compared to those women in the poorest quintile category.

Religious affiliation was significantly associated with the odds of PPFP utilization in both Burundi and Rwanda. Table 2 shows that in both Burundi and Rwanda, protestant women experienced reduced odds of PPFP compared to Catholics, the reference category. Protestant women in Burundi had 0.8 times (95% CI=06-0.9) the odds of PPFP utilization compared to Catholics, while in Rwanda the former category of women had 0.7 the odds (95% CI=0.6-0.8) of PPFP compared to the reference category. Whereas Moslem and other religious groups did not depict significant model findings in Rwanda, for Burundi these categories were significant. Moslem women in Burundi experienced 1.5 times (95% CI = 1.1-2.3) the odds of PPFP utilization, while other categories of religion experienced 2 times (95% CI = 1.3-3.7) the odds of PPFP utilization. Women who delivered with assistance of a qualified health professional were 1.2 times (95% CI = 1.0-1.4) more likely to utilize PPFP in Rwanda, compared to those who were delivered by other means. This later relationship was not significant for women in Burundi. In addition residence in rural areas was associated with reduced odds of PPFP in Burundi (OR=0.8; 95% CI = 0.6-1.0). In Rwanda, rural urban differences in PPFP were not significant in the regression equation. With regard to exposure to media, women who responded in the survey that they had been exposed to media were significantly more likely to utilize PPFP compared to those who had no such exposure. In Burundi, women who had media exposure were 1.6 times more likely to utilize PPFP (95% CI = 1.3-1.9), while in Rwanda women with media exposure had 1.3 times the odds (95% CI = 1.2-1.3) of utilizing PPFP compared to those who had no exposure. Finally, an increase in the number of living children was directly associated with PPFP utilization in both Burundi and Rwanda, respectively. The finding presented in Table 2 show that the odds of utilizing PPFP increased by nearly 30%, in both Burundi and Rwanda with an additional living child for a woman.

## Discussion

The objectives of this paper were twofold-to establish the level of contraceptive use during postpartum period in Burundi and Rwanda, and to analyze the predictors of PPFP in the two study populations. The findings show that PPFP utilization was higher in Rwanda compared to Burundi. The reasons for high family planning utilization in Rwanda compared to Burundi could be explained by major improvements in Rwanda's health system, infrastructure and social programs including male involvement in reproductive health issues [20]. In Burundi on the other hand evidence suggests that there a strong cultural and societal pressures on women to bear more children [21]. Decision-making on contraceptive use is the shared responsibility of men and women and effective development and implementation should address barriers to men's supportive participation in reproductive health, including addressing men's negative beliefs regarding contraceptive services [22]. From the analysis, the factors that predicted PPFP utilization in Burundi and Rwanda revealed both similarities and differences. First, the relationship between women's education and PPFP utilization appears to be universal and suggests a direct association [7, 14, 23-25]. This relationship could be a reflection of women autonomy and decision making roles that invariably tend to shift with increasing level of education. Whereas wealth status was an important factor determining PPFP in Rwanda, it was not the case with Burundi. The relationship between the wealth status indicator and PPFP utilization to a greater extent is related to both financial autonomy and access to resources necessary to purchase the required health inputs [7, 26]. Further in Rwanda, an equity analysis of performance based financing in Rwanda, showed increased utilization of services at health facilities across wealth status [20, 27, 28, 29]. Among the few studies conducted in both countries, one showed wealth status as a significant factor in influencing pregnancy history and current use of contraception in Rwanda and Burundi [30].

There is limited research to provide plausible explanations for the low postpartum family planning utilization. The low use could be attributed to the prolonged civil unrest in Burundi with tremendous impact on service utilization [31]. In a Nepalese study, women within the poorest wealth quintile were found to exhibit a high unmet need for family planning during the postpartum period [10]. We observe that uptake of PPFP was significantly lowest among Protestant women compared to Catholics both in Burundi and Rwanda, respectively. This finding is similar to the one found in Uganda [7], however the latter study offered no explanation for this phenomenon. One plausible explanation for this finding could be the Protestant pronatalist religious ethic, which in a way has blended in the high fertility culture. Nonetheless, this is one area that would require further investigation and explanation possibly using qualitative approaches. This study also showed that in Rwanda, the rural urban differences are not significant as was the case in Burundi and indeed in other African countries. The ability to bridge the rural urban differences in availability and provision of reproductive health services in Rwanda is likely to have contributed to the observed positive PPFP utilization result. We observed that exposure to media increased the uptake of PPFP for both Burundi and Rwanda. It is also clear that the media has a crucial role to play in demographic and health transition in African countries. This finding is consistent with results from a previous study in Kenya, which suggested that the mass media can have an important effect on reproductive behavior [32]. Mass media campaigns are widely used to expose high proportions of large populations to messages through routine uses of existing media, such as television, radio, and newspapers [33]. Exposure to such messages, though passive has potential to impact behaviour change in the long-term.

Finally, a unit increase in the number of children born to a woman significantly increased the likelihood of PPFP utilization for both Burundi and Rwanda. At the same time increase in the age of the woman was associated with a reduction in her likelihood of utilizing PPFP for the two study populations. The findings concerning age of woman and number of children ever born correspond to those of a similar study in Uganda [7]. The likely explanation for this finding is that PPFP is more prevalent among younger women (< 25 years), who at the same time are more receptive to family planning methods compared to older women [34]. The findings from our study have important policy implications. Our study highlights the need for governments of Burundi and Rwanda to address gaps in the provision of PPFP. The results suggest that programs aimed at enhancing postpartum contraceptive use should target women with low education, low wealth status and that the media has an important role to play in this transformation. Policies and programs must be put in place to ensure that the rural urban differences are eradicated in Burundi as is the case in Rwanda.

**Study limitations:** The cross-sectional nature of the data poses causality challenges. It is difficult to ascertain the association between PPFP and the predictor variables since they were measured at one point in time. The study did not address all health system related factors that affect postpartum family planning utilization. Notably PPPFP is often related to the quality of counseling received by mothers during the post delivery period [33, 34]. Despite these limitations, we used reliable data and appropriate methods hence the findings reflect accurately on PPFP utilization in Uganda. The large size of this study and its likely representativeness is a great strength.

## Conclusion

Our study findings point to the potential issues to address in order to improve delivery of PPFP and family planning in general in the two East African countries. Interventions to enhance postpartum contraceptive use should target women with low education, low wealth status and that the media has an important role to play in this transformation. Policies and programs must be put in place to ensure that the rural urban differences are eradicated.

### What is known about this topic

- Low use of contraception among post part partum women remains an impediment to reducing fertility in low income countries;
- Sub-Saharan Africa, has one of the highest rates of none use of contraception in the post-partum period.

### What this study adds

- The importance of addressing socio-economic factors including, low wealth status, low education and media in enhancing post-partum contraceptive use in a sub-Saharan setting; these factors once addressed, they do have a great role to play in the transformation of people's lives in the two countries;
- The cost of having women with no education remains health burden since they are more likely to have mistimed births, unwanted pregnancies, child related illness and mortality;
- Addressing and provision of country specific interventions that would promote the utilization postpartum contraception. Well-designed community based interventions reaching out to all women in wound enhance the utilization of contraceptives among different population groups.
